# Use of contrast-enhancement and high-resolution 3D black-blood MR Imaging to identify inflammation in rabbit atherosclerotic plaques

**DOI:** 10.1186/1532-429X-13-S1-P22

**Published:** 2011-02-02

**Authors:** Yoo Jin Hong, Jin Hur, Jaeseok Park, Young Jin Kim, Hye-Jeong Lee, Byoung Wook Choi, Kyu-Ok Choe

**Affiliations:** 1 Severance hospital, Seoul, Korea, Republic of

## Background

Inflammation plays a critical role in plaque initiation, progression, and disruption. As such, inflammation represents an emerging target for the treatment of atherosclerosis.

## Purpose

We investigated the contributing factors for plaque enhancement and examined the relationships between regional contrast enhancement and the inflammatory activity of atherosclerotic plaques in an experimental rabbit model using contrast-enhanced high-resolution 3D black-blood magnetic resonance imaging (MRI) in comparison with histopathology.

## Methods

Ten atherosclerotic rabbits and three normal control rabbits underwent high-resolution 3D contrast-enhanced black-blood MRI. MR images and the corresponding histopathological sections were divided into four quadrants. Plaque composition was analyzed for each quadrant according to histopathological (percent of lipid-rich, fibrous, macrophage area and microvessel density) and imaging criteria (enhancement ratio (ER), ER=SIpost/SIpre).

## Results

A total of 62 non-calcified plaques (n=248, 156 lipid-rich quadrants and 92 fibrous quadrants) were identified based on histopathology. Mean ER values were significantly higher in atherosclerotic vessel walls than in normal vessel walls (2.03 ± 0.25 vs 1.58 ± 0.15, p = 0.017). Mean ER values were significantly higher in macrophage-rich plaques compared to the macrophage-poor plaques (2.21 ± 0.28 vs 1.81± 0.22, p = 0.008). Using multiple regression analysis, macrophage area and microvessel density were independently associated with ER values that reflected plaque enhancement (p <0.001).

## Conclusion

Contrast-enhanced high-resolution 3D black-blood MRI may be an efficient method to predict plaque inflammation.

